# A fabric-based hydrovoltaic electricity generator with multi-component carbon black for sustainable energy output†

**DOI:** 10.1039/d4ra02346c

**Published:** 2024-06-12

**Authors:** Yahua Liu, Bingzhong Xiao, Quanmao Wei, Zichao Yuan, Wenzhuo Song, Ling Zhou, Wenna Ge

**Affiliations:** a State Key Laboratory of High-performance Precision Manufacturing, Dalian University of Technology Dalian 116024 China yahualiu@dlut.edu.cn

## Abstract

Water evaporation-induced electricity generators are considered a promising green energy-harvesting technology to alleviate the increasingly serious fossil energy crisis. Previous water evaporation-induced electricity generators mainly focused on single component carbon black, limiting the improvements in energy output. At present, there are relatively few studies on multi-component carbon black for improving electricity-generation performance. Herein, inspired by plant transpiration, we designed a fabric-based water evaporation-induced electricity generator (FWEG) based on multi-component carbon black, which can maintain a voltage of 0.65 V for more than 48 h. Through the synergistic effect of multi-component carbon black-enhanced oxygen-containing functional density, the FWEG can generate an enhanced output current of 61.61 μA without any additional energy input. Moreover, we show that the FWEG can be integrated readily to charge commercial capacitors or directly power LED lights and calculators for a long time. This work provides new insights for designing high-performance hydrovoltaic electricity generators for sustainable green energy harvesting.

## Abstract 2

Multi-component carbon black enhanced oxygen-containing functional density through a synergistic effect. The FWEG could generate electricity without any additional energy input and could be readily integrated to directly power calculators.

## Graphical abstract

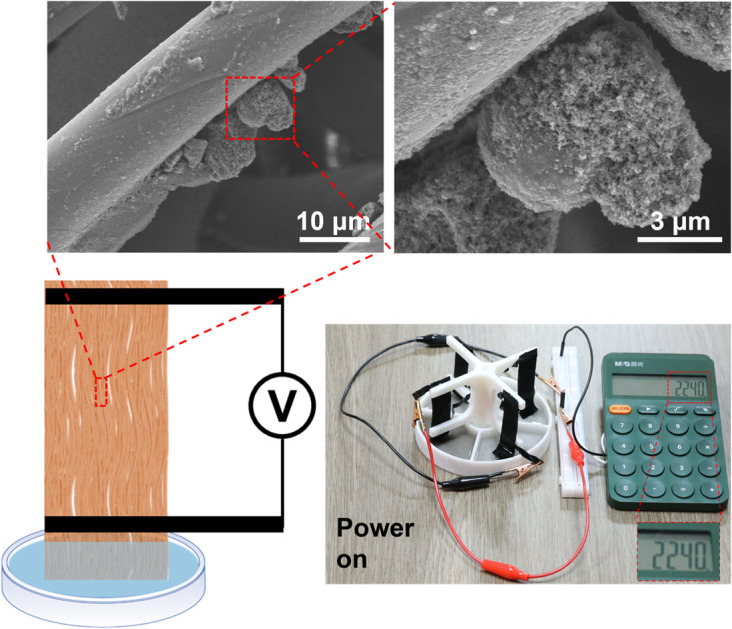

## 1 Introduction

With the increasingly severe fossil energy crisis and global warming, harvesting green and clean energy has become one of the most promising solutions to achieve carbon neutralization and meet the ever-increasing electricity demands.^1,2^ In recent years, various strategies for green energy harvesting, including photovoltaic electricity generation,^3–6^ piezo-/triboelectric nanogenerators,^7–11^ and thermoelectric nanogenerators,^12–14^ have been proposed, but these are still restricted by brief pulse signal or specific environmental requirements. In this regard, water evaporation-induced electricity generation has been attracting significant attention owing to the abundance, cleanliness, and sustainability of water.^15^

Since its advent in 2017, the majority of water evaporation-induced electricity generators (WEGs) were fabricated from expensive materials (graphene, CNTs, silicon wafers, *etc.*), which limited their practical application. Recently, low-cost carbon back has been introduced to design WEGs for sustainable green energy transformation.^16^ However, WEGs with single component carbon back only produce a low ion concentration in water due to their limited solid–liquid contact area, resulting in low current output. At present, various strategies for enhancing current performance have been developed, including changing solution properties^17^ and device shape^18–20^ and enhancing surface functional groups,^16,21,22^ but some corresponding problems persist, such as salt accumulation, inconvenient large size, and complex preparation process.

Plants represent efficient evaporation systems. Through transpiration,^23–25^ water is absorbed through the roots of plants, transported *via* the stems through capillary action and water potential differences, and is finally evaporated from the surface of the leaves. Inspired by the water-evaporation process in plants, we designed a fabric-based water evaporation-induced electricity generator, which is fabricated using carbon black particles with different particle sizes assembled onto the surface of non-woven fabrics through simple dip-coating and blade-coating techniques. The non-woven fabric under an asymmetric wetting gradient can absorb water from one end, transport it to the other end *via* capillary effect, and evaporate it during the transport. The density of oxygen-containing functional groups required for electricity generation was increased by the synergistic effect of multi-component carbon black, and the electricity-generation performance was hence improved. Under optimized conditions, the FWEG could still maintain a high electrical-power-generation performance with an open-circuit voltage (*V*_OC_) of 0–0.65 V and a short-circuit current (*I*_SC_) of 0–61 μA for more than an hour under ambient conditions (temperature 25–28 °C, and humidity 50–60%). Integrating FWEGs could directly power commercial electrical devices, such as LEDs and calculators. Moreover, a 1000 μF commercial capacitor could also be easily charged to more than 1.5 V within 50 s. We believe that this FWEG can provide a promising way to obtain long-term maintenance-free high electricity generation from green sustainable energy.

## 2 Experimental section

### 2.1. Materials and chemicals

N330 carbon black (about 1250 mesh) was purchased from http://taobao.com/ (Ruishan Trade). C325 carbon black (particle size about 50 nm) was obtained from http://taobao.com/ (Tianjin Huacai Chemical Co., Ltd). Sodium dodecyl benzene sulfonate (SDBS) was purchased from Sinopharm Chemical Reagent Co., Ltd. Polyvinyl alcohol (PVA) was obtained from Aladdin. Polypropylene (PP) spun-laced non-woven fabric was bought from http://taobao.com/ (Jiada non-woven fabric). Conductive tape was purchased from http://taobao.com/ (Rigorous). The deionized water was produced using an ultra-pure water machine (Summer-S2-20H, Sichuan Delishi Technology Co., Ltd, China) and was used in all the experiments, unless otherwise specified.

### 2.2. Fabrication of carbon black ink

First, 1 g carbon black and 0.5 g SDBS were added to 40 g deionized water and dispersed uniformly by ultrasonic equipment. Different proportions of carbon black inks were applied with varying weight ratios of the two carbon blacks by controlling the total amount of carbon black and naming the resulting inks according to the corresponding ratio. For example, the ratio N10 represents the ink prepared from 1 g N330 carbon black. The ratio of N7C3 represents the ink prepared by the weight ratio of N330 to C325 of 7 : 3 and the total weight of 1 g. The remaining carbon black inks were named N5C5 ink, N3C7 ink, and C10 ink in the same way.

### 2.3. Instruments and characterization

The carbon black ink was dispersed by an ultrasonic cleaner (KQ-250DE, Supmile, Kunshan Ultrasonic Instrument Equipment Co., Ltd). The microstructures and element distribution of the generators were characterized by field emission scanning electron microscopy (FESEM, JSM-7900F Plus, JEOL, Japan). The surface functional groups of carbon black were analyzed by advanced Fourier transform infrared spectroscopy (FTIR, 6700, ThermoFisher, USA). The zeta potential of the carbon black ink was measured using a zeta potential analyzer (Zeta sizer Nano ZS90, Malvern, UK). The open-circuit voltage (*V*_OC_) and short-circuit current (*I*_SC_) of the generator were recorded using an electrochemical workstation (ECW, CHI660E, Chenhua, China).

## 3 Results and discussion

### 3.1. Fabrication and characterization of the FWEG

**Fig. 1:**
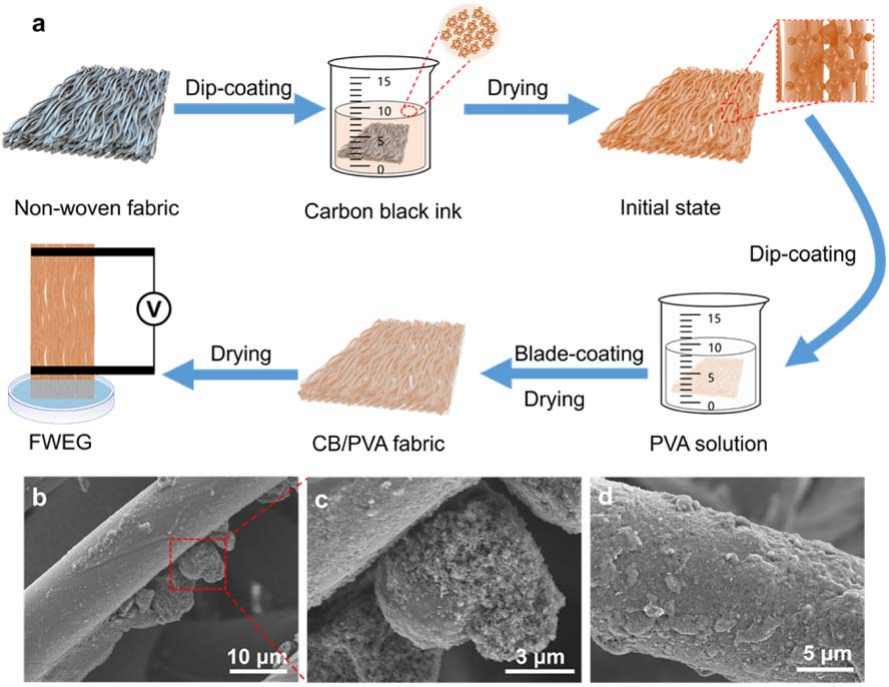
Fabrication and characterization of the FWEG. (a) Fabrication of the FWEG. (b) SEM images of carbon black on the surface of the fabric. (c) Adhesion of C325 carbon black on the surface of N330 carbon black. (d) SEM image of the surface after dipping in PVA aqueous solution.

In the manufacturing process of the FWEG, as shown in Fig. 1a, we chose non-woven fabric as the base material due to its flexibility, good strength, and rich fabric, so that it would have a rich surface for the attachment of carbon black particles and provide good structural support for the water flow. The manufacturing process mainly adopted a simple dip-coating process. First, the commercial non-woven fabric was cut into a rectangle with a size of 1.5 cm × 6 cm, and was then dipped into the prepared carbon black ink. After the dip-coating process, we obtained the attached carbon black particles on the fabric surface. The mass change of the electricity generator with the attached carbon black particles reflected the loading of carbon black, and different carbon black loads could be adjusted by varying the dip-coating times of the electricity generator. Subsequently, the initially dried electricity generator was immersed in 10 wt% PVA aqueous solution and bladed by a blade-coating machine to remove the excess PVA aqueous solution on the surface of the electricity generator, with an aim to avoid the additional impact on the sample due to too much or too little PVA aqueous solution dip-coating. The dip-coating of PVA aqueous solution could improve the problem of an insufficient bonding strength between the carbon black and non-woven surface, improve the stability of the electricity generator, and allow obtaining the CB/PVA fabric after drying. Finally, two conductive tapes with a width of 0.5 cm were used as electrodes to connect the two ends of the CB/PVA fabric, and the distance between the two electrodes was 3 cm to obtain the FWEG.

The SEM image of the original commercially available non-woven fabric showed that the fabric was arranged in a very orderly manner and the surface of the fabric was smooth (Fig. S1†), which was conducive to carbon black adhesion. In this work, by adhering small carbon black C325 to the surface of the large N330 (Fig. 1b and c), the density of the oxygen-containing functional groups on the fabric was significantly increased to improve the electricity-generation performance. Meanwhile, small carbon black can easily cause internal short circuits while large carbon black can lead to excessive internal resistance, which could be effectively solved through the synergistic combination of multi-component carbon black.^26,27^ The attachment of PVA to the outermost layer (Fig. 1d) can reduce the loss of carbon black particles from the surface of the non-woven fabric. EDS analysis confirmed the large distribution of carbon elements on the surface of the nonwovens (Fig. S2†).

The zeta potential of the N3C7 carbon black ink was measured as −32.0 mV (Fig. S3†), indicating that the surface of the carbon black particles was negatively charged after exposure to water. At the same time, the FTIR spectra and XPS spectra of the electricity generator (Fig S4 and S5†) proved that the carbon black was rich in oxygen-containing functional groups (–OH, –COOH),^28–35^ which dissociate after contact with water to produce positively charged H^+^ and a negatively charged surface, which is an important process for water evaporation-induced electricity generation.

### 3.2. Optimization and evaluation of the FWEG

**Fig. 2:**
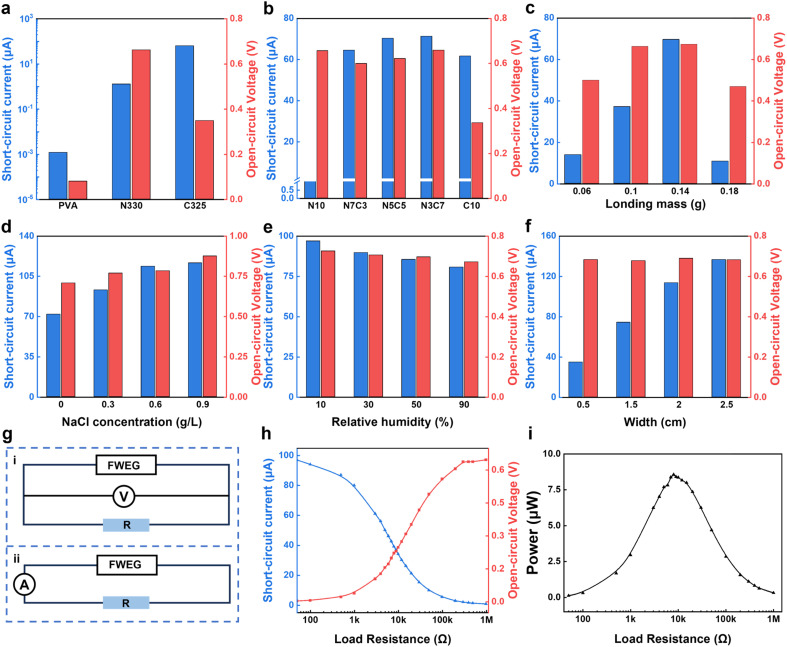
Optimization and evaluation of the electricity-generation performance. (a) Power-generation-activity test of carbon black. (b) Electricity-generation performance of different carbon black inks. (c) Electricity-generation performance of different carbon black loads. (d) Electricity-generation performance of different liquids (water, 0.3 g L^−1^ NaCl, 0.6 g L^−1^ NaCl, and 0.9 g L^−1^ NaCl). (e) Electricity-generation performance of different relative humidities. (f) Electricity-generation performance of different widths. (g) Circuit diagram for testing the electricity-generation performance at different loads: (i) open-circuit voltage test circuit diagram and (ii) short-circuit current test circuit diagram. (h) Voltage and current performances under different load resistors. (i) Electricity-generation performance of different load resistors.

In order to obtain a FWEG with excellent electricity generation performance, the important parameters in the generator manufacturing process were investigated (Fig. 2). This included the electricity-generation activity of carbon black, the composition of the carbon black ink, and the loading capacity of carbon black. First, the open-circuit voltage (*V*_OC_) and short-circuit current (*I*_SC_) of the electricity generator were measured using an electrochemical workstation. The size of the fixed electricity generator was 1.5 cm × 6 cm, the distance between electrodes was 3 cm, and the carbon black weight of the electricity generator loaded with carbon black was 0.14 g.

In order to ensure the role of carbon black in the electricity-generation process (Fig. 2a), we compared the open-circuit voltage and short-circuit current of three electricity generators with different components, *i.e.*, only 10 wt% PVA, N330/PVA, and C325/PVA. It was found that the voltage and current of the electricity generator made only of the non-woven fabric and 10 wt% PVA aqueous solution were almost zero, while the other two electricity generators had corresponding voltage and current values, which shows that carbon black plays an indispensable role in the electricity-generation, and excludes the influence of other factors on electricity generation.

By testing the generators prepared with five carbon black inks, the influence of different carbon black composition ratios on the electricity-generation performance under the synergistic effect of the multi-component carbon black was studied (Fig. 2b). With the increase in the ratio of C325 carbon black, the current first increased and then slightly decreased, while the voltage fluctuated and then decreased. Therefore, the optimal value was obtained when the ratio was N3C7. The reason is that when the ratio was C10, the carbon black particle size of C325 was small, resulting in strong conductivity, reduced carbon black layer resistance, and an increased likelihood of internal short circuits,^36^ thereby affecting its electricity generation performance. When the ratio was N10, the output performance was also affected by its large particle size and relatively weak conductivity. Furthermore, the optimal ratio of N3C7 was selected to prepare the electricity generator with different carbon black loadings.

With the increase in carbon black loading (Fig. 2c), it was found that the initial voltage and current increased, and finally reached a steady state voltage of 0.65 V and current of 61.61 μA. Initially, with the increase in carbon black load, the electricity-generation performance was improved, because the increase in carbon black loading increased the density of oxygen-containing functional groups, facilitating dissociating enough ions to meet the electricity generation after contact with water, and generating negatively charged channels, which was conducive to the improvement of the electricity-generation performance. However, when the loading of carbon black continued to increase to 0.14 g or more, the electricity-generation performance decreased, because too many carbon black particles can affect the original channel structure and make it more prone to internal short-circuit problems. In general, the optimized parameters of the water evaporation-induced electricity generator included a carbon black ratio of N3C7 and carbon black weight of 0.14 g.

In addition, the electricity-generation performance of the FWEG was evaluated under different liquids, relative humidity, and width variation. When the water evaporation-induced electricity generator generated electricity with water and 0.9% NaCl solution (Fig. 2d), the *V*_OC_ and *I*_SC_ increased from 0.708 V to 0.876 V and from 71.9 μA to 116.6 μA, respectively, because the salt solution contained more ions than deionized water did. The internal resistance of the active material for electricity generation and the thickness of the electrical double layer (EDL) also change, which will affect the electricity-generation performance. The increase in ion concentration reduces the internal resistance, which is beneficial to the electricity-generation. The thickness of the EDL decreased with the increase in ion concentration, which enhanced the ion-selective permeability and charge density and improved the electricity-generation performance.^36^ In addition, the increase in salt concentration increased its surface tension.^35^ Under the action of capillary force, it was more conducive to water movement and would also have a positive effect on the electricity-generation performance.

At room temperature of 25 °C, increasing the relative humidity from 10% to 90% hurt the power-generation performance (Fig. 2e), reducing the *V*_OC_ and *I*_SC_ by 0.054 V and 16.29 μA, respectively. The evaporation process slowed down when the relative humidity was high, and the movement of water was hindered, which had a certain impact on the electricity-generation performance.^37^ By fixing the length of the electricity generator at 6 cm, the influence of the width change on the output performance was studied (Fig. 2f). It could be observed that as the width increased from 0.5 cm to 2.5 cm, the *I*_SC_ also increased from 34.67 μA to 136.58 μA, while the *V*_OC_ was stable. This is due to the increase in width, which could be considered as equivalent to multiple electricity generators with their respective positive electrodes connected, and their respective negative electrodes also connected, equivalent to the parallel connection of the equipment.^38^ The generator generation power was tested by connecting load resistors with different resistance values, and by the corresponding circuit diagram (Fig. 2g). As the load resistance increased from 50 Ω to 1 MΩ, the voltage at both ends of the load resistance increased from 0.0013 V to 0.64 V, while the current in the series loop decreased from 97 μA to 0.5 μA (Fig. 2h). The generation power could be calculated as:

1 *P* = *U* × *I*

The output power increased with the load resistance until the load resistance reached 8000 Ω, but then decreased subsequently with further increasing the load resistance (Fig. 2i). With a peak output power of up to 8.57 μW, the inner resistance of the FWEG was consistent with the optimum load resistance.^39,40^

### 3.3. Electricity-generation mechanism of the FWEG

**Fig. 3:**
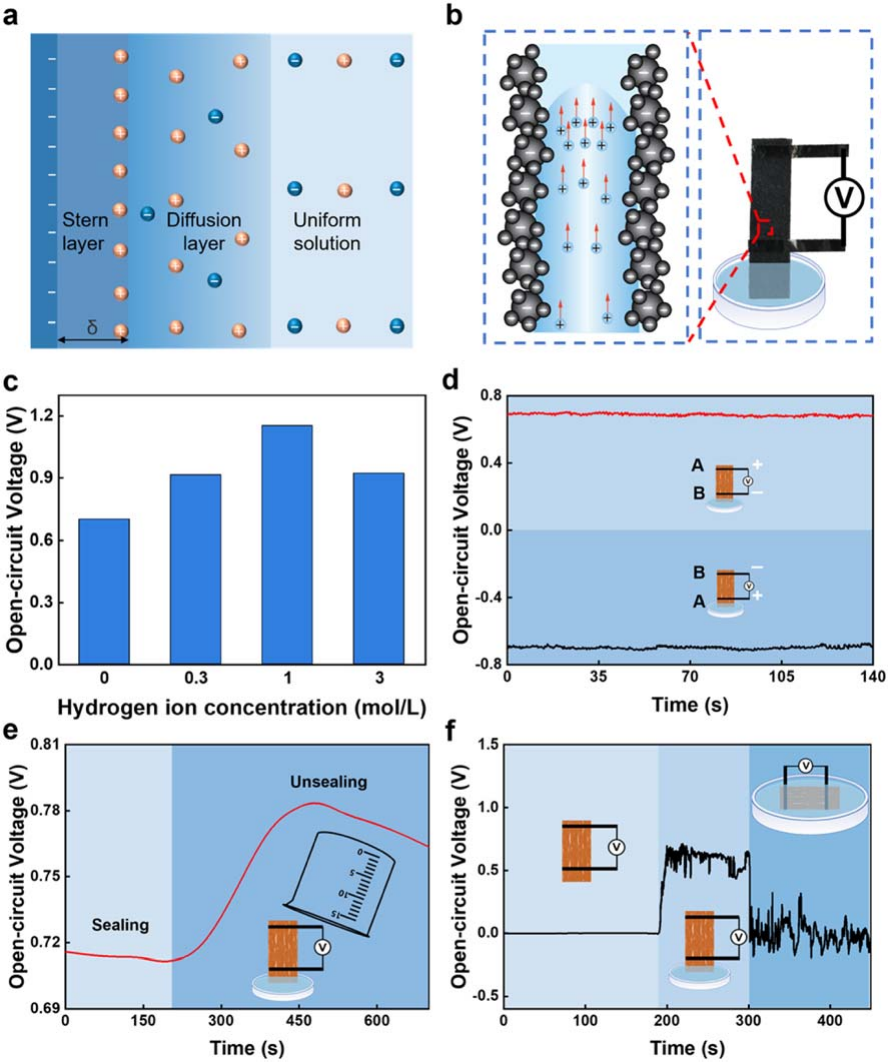
Electricity-generation mechanism. (a) Electric double layer principle. (b) Schematic diagram of the power-generation principle. (c) Voltage of the generator under different hydrochloric acid concentrations. (d) Switching polarity experiment for the device. (e) Voltage under sealed and unsealed conditions, and (f) different degrees of immersion in the water experiment.

Based on the above results, the electricity generation mechanism of the FWEG is proposed (Fig. 3). The generation of electricity can be attributed to the streaming potential.^16^ Specifically, when the bottom of the FWEG is in contact with water, the functional groups interact with the water molecules to dissociate H^+^. The remaining immovable functional groups spontaneously form the EDL^41^ on the carbon black surface (Fig. 3a), which can be proved by the measurement of the zeta potential (Fig. S3†). The negatively charged channel repel the ions with the same electrical properties in the water, while the ions with opposite electrical properties (H^+^) can pass normally. Therefore, H+ moves upward along with the water flow and accumulates at the top electrode, inducing a potential difference and current (Fig. 3b).

To verify the connection between H^+^ and evaporation and the dynamo mechanism, further research was carried out. The concentration of hydrochloric acid represents the concentration of H^+^ (Fig. 3c). The test results show that, with the increase of H^+^ concentration, the electricity-generation performance of the generator was improved, which proved the important role of H^+^ in the electricity-generation.^40^ Among the tested parameters, the decrease in voltage was due to the influence of excessive H^+^ concentration on the EDL.

To confirm the evaporation induced the voltage, first we tested when the device electrode was turned over and the other end was dipped into water. We found that the output voltage could reach the same amplitude (Fig. 3d),^16^ while the voltage symbol was the opposite, proving that the output of electric energy was induced by water evaporation. In addition, the experimental system was sealed in a beaker (Fig. 3e). The conversion for sealing and unsealing the experimental system caused a significant change in *V*_OC_, indicating that water evaporation was indeed one of the driving forces for the electric energy generation. However, the voltage did not eventually drop to zero after a long period of sealing the system. This indicates that water evaporation was not the only driving force for electricity generation, and may also include the capillary force of the device itself.^40^ After that, we tested the electricity-generation performance of the generator in different degrees of immersion in water (Fig. 3f). It was observed that the generator had no voltage output when there was no water contact or was completely immersed in water. This shows that the electricity generator will produce a potential difference under the asymmetric conditions of the dry and wet regions,^17^ because the power-generation process needs water evaporation, and does not evaporate without contact with water or when completely immersed in water, resulting in zero voltage.

### 3.4. Application prospects of the FWEG

**Fig. 4:**
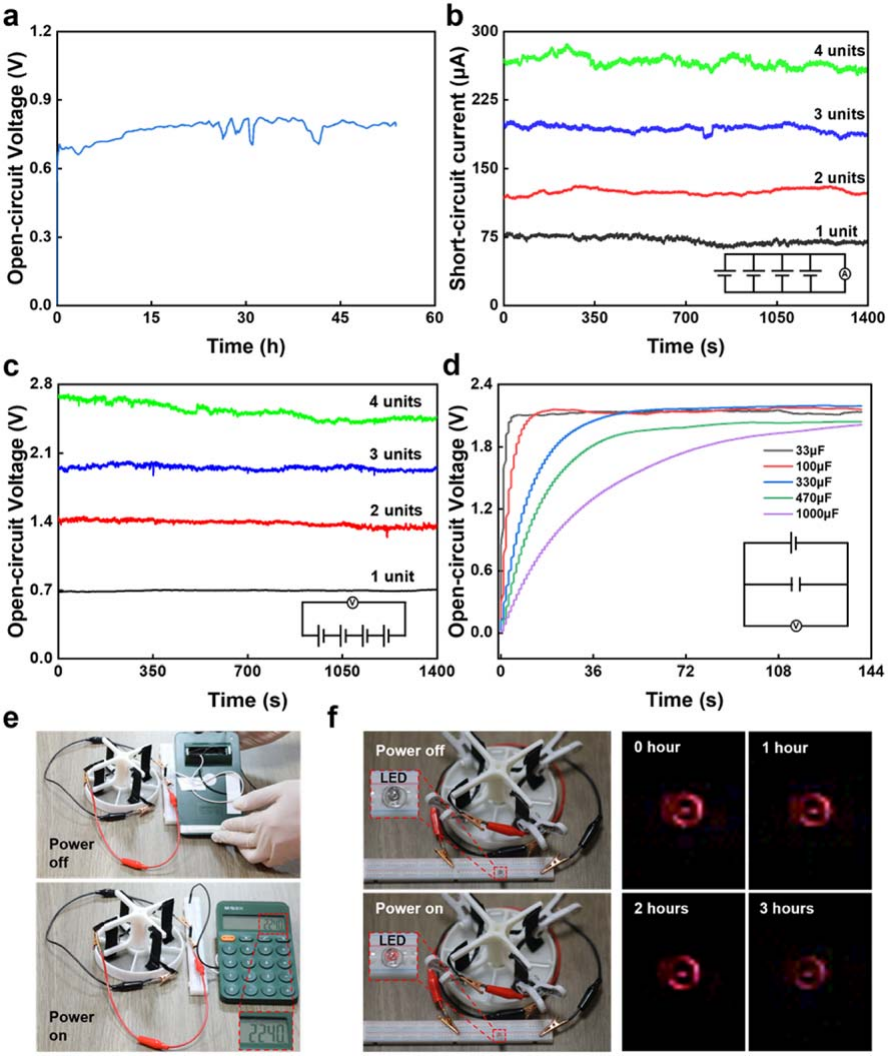
Generator applications. (a) Open-circuit voltage for long-term electricity generation. (b) Short-circuit current for four generators in parallel. (c) Open-circuit voltage along with time for four generators in series. (d) Charging commercial capacitors. (e) Powering a calculator. (f) Powering an LED.

The generator applications are related to continuous power generation, integration, and commercial equipment power supply (Fig. 4). In the experiment, a single generator could continuously generate electricity for more than two days (Fig. 4a and S6†). By paralleling four electricity generators, the *I*_SC_ could be increased from 0 μA to 260 μA (Fig. 4b). When the four electricity generators were connected in series, the *V*_OC_ could reach 2.4 V, and the voltage was close to 4 times that of a single electricity generator (Fig. 4c). Commercial capacitors of 33, 100, 330, 470, and 1000 μF could be charged to more than 1.5 V in a short time through the four electricity generators in series (Fig. 4d). In addition, the four series-connected electricity generators could directly supply power to a calculator (Fig. 4e and Video S1†). Furthermore, they could directly supply power for a commercial LED without the participation of capacitors, and the power supply time could reach 3 h (Fig. 4f and Video S2†).

## 4 Conclusions

In summary, inspired by the transpiration of plants, our work demonstrates a flexible water evaporation-induced electricity generator based on fabric materials. A high functional group density was the key to improving the power-generation performance of the generator. In this work, the density of oxygen-containing functional groups in the generator was increased in cooperation with multi-component carbon black, and the generator could deliver a continuous and high electrical output of ∼0.65 V and ∼61.61 μA through water transportation in a low-cost fabric. By connecting four electricity generators in series, it could power LED lights or a calculator for a long time and charge commercial capacitors. Our work shows great prospect of using water evaporation to realize green renewable energy generation and provides important ideas for alleviating the current energy crisis.

## Author contributions

The manuscript was written through contributions of all authors. All authors have given approval to the final version of the manuscript.

## Conflicts of interest

There are no conflicts to declare.

## Supplementary Material

RA-014-D4RA02346C-s001

RA-014-D4RA02346C-s002

RA-014-D4RA02346C-s003
