# Supplementary material for: A fabric-based hydrovoltaic electricity generator with multi-component carbon black for sustainable energy output
Source: RSC Adv. 2024 Jun 12;14(26):18832–7. doi: 10.1039/d4ra02346c (PMC11167618; doi:10.1039/d4ra02346c)
Supplement: RA-014-D4RA02346C-s001 [file RA-014-D4RA02346C-s001.pdf]

Electronic Supplementary Material (ESI) for RSC Advances.
This journal is © The Royal Society of Chemistry 2024

Supplementary information

A fabric-based hydrovoltaic electricity generator with multi-component carbon black for sustainable energy output

Yahua Liu, *[a] Bingzhong Xiao [a], Quanmao Wei [a], Zichao Yuan [a], Wenzhuo Song [a], Ling Zhou [a], Wenna Ge*[a]

[a] Key Laboratory for Precision & Non-traditional Machining Technology of Ministry of Education, Dalian University of Technology, Dalian 116024, China.
E-mail: yahualiu@dlut.edu.cn.

Supplementary Videos

Supplementary video 1. Four FWEGs in series to drive an LED for 3 hours.

Supplementary video 2. Demonstration of four generators in series to power the calculator.

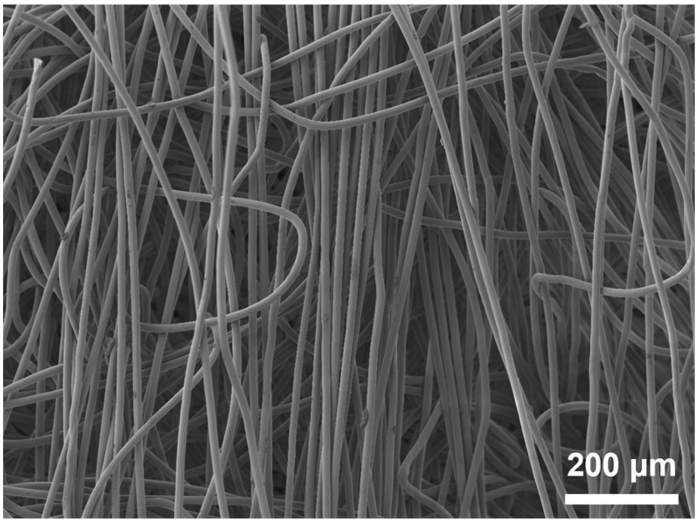

Fig. S1: SEM images of the original commercially available non-woven fabric.

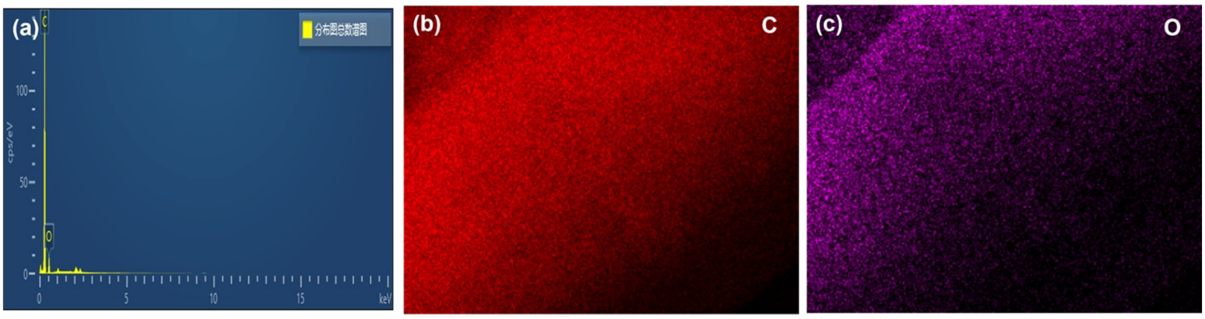

Fig S2: EDS image of FWEG. (a) Total spectrum of distribution map. (b) Carbon element distribution. (c) Oxygen distribution.

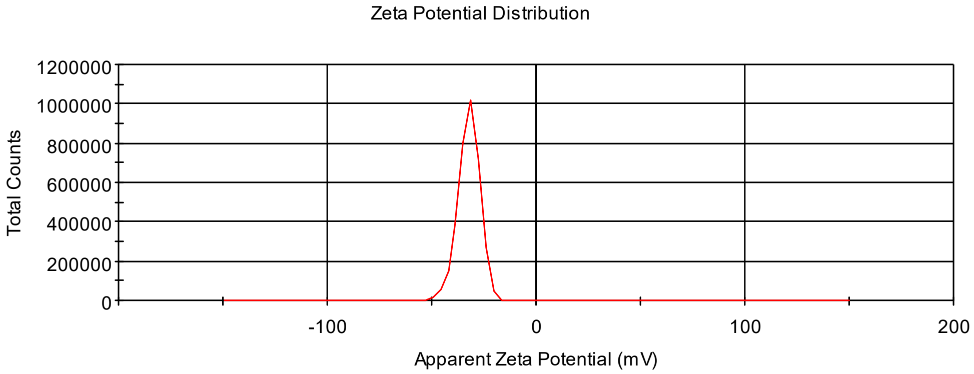

Fig S3: Zeta potential of N3C7 Carbon black.

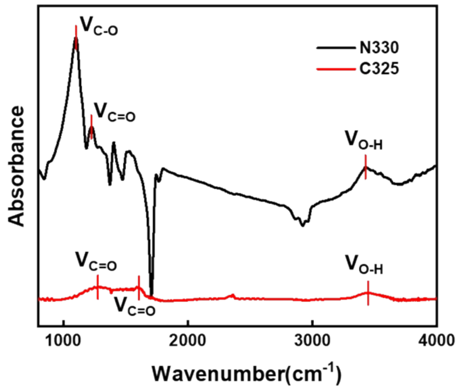

Fig S4: Fourier transform infrared spectrum (FTIR) of carbon black. N330 carbon black produces characteristic peaks of infrared spectrum at 1098 cm^{-1}, 1220 cm^{-1} and 3421 cm^{-1}, and C325 carbon black produces characteristic peaks of infrared spectrum at 1278 cm^{-1},1597 cm^{-1} and 3438 cm^{-1}, which proved that Carbon black was rich in oxygen-containing functional groups (-OH, -COOH).

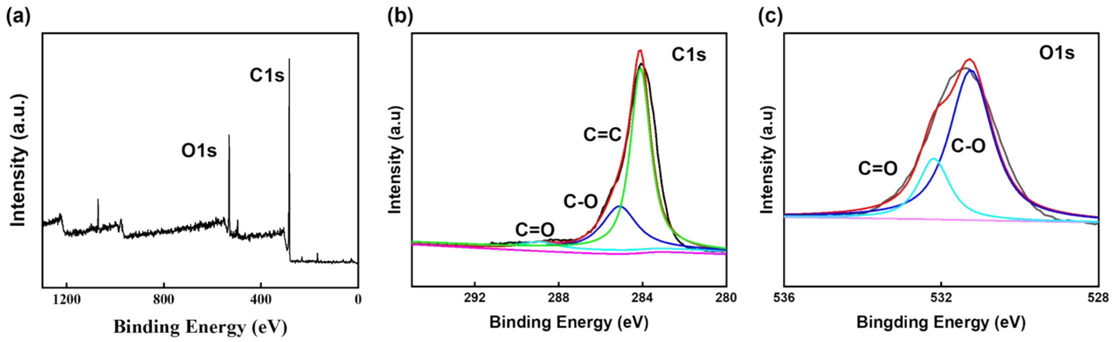

Fig S5: XPS spectra of N3C7 Carbon black.

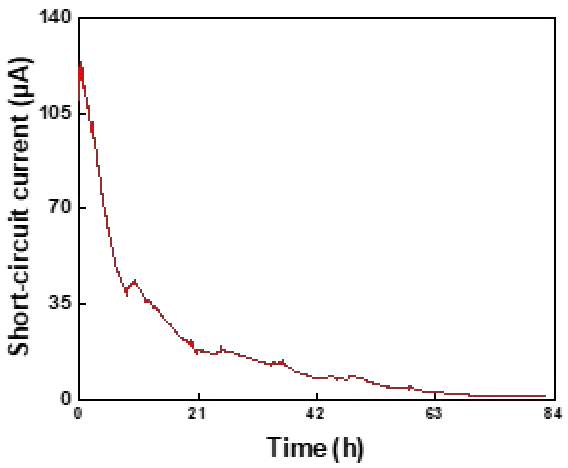

Fig S6. Short-circuit current testing for long-term electric generation.

Table 1. Comparation of the performances and cost of relevant researches

Materials	Current density (μA/cm^2)	Cost (RMB/g)	Ref.
Al_2O_3	0.01111	1.764	1
MoS_2	0.01389	12.43219	2
TiO_2	0.01511	1615.735	3
$AlCl_3$	0.049	5.7204	4
Wood	0.02067	0.04	5
PAN	0.92125	19.143	6
MWCNT	1.51111	333.022	7
Graphite	0.02967	0.49173	8
SiO_2	0.0381	0.48	9
PPy	2.66667	199.9	10
Carbon black	8.54444	0.0208	This work

References

1. C. Shao, B. Ji, T. Xu, J. Gao, X. Gao, Y. Xiao, Y. Zhao, N. Chen, L. Jiang and L. Qu, *ACS Appl Mater Interfaces*, 2019, **11**, 30927-30935.
2. S. G. Yoon, H. D. Jin, W. H. Lee, J. Han, Y. H. Cho and Y. S. Kim, *Nano Energy.*, 2021, **80**, 105522.
3. B. X. Ji, N. Chen, C. X. Shao, Q. W. Liu, J. Gao, T. Xu, H. H. Cheng and L. T. Qu, *J Mater Chem A.*, 2019, **7**, 6766-6772.
4. Q. Ma, Q. He, P. Yin, H. Cheng, X. Cui, Q. Yun and H. Zhang, *Adv Mater*, 2020, **32**, e2003720.
5. J. Zou, W. Fang, X. He, H. Chen, X. Du, T. T. Jiang, J. P. Qin and L. Zhao, *Journal of Materials Science*, 2021, **56**, 16387-16398.
6. T. Tabrizizadeh, J. Wang, R. Kumar, S. Chaurasia, K. Stamplecoskie and G. Liu, *ACS Appl Mater Interfaces*, 2021, **13**, 50900-50910.
7. R. Kumar, T. Tabrizizadeh, S. Chaurasia, G. J. Liu and K. Stamplecoskie, *Sustainable Energy & Fuels*, 2022, **6**, 1141-1147.
8. L. Wang, L. Liu and N. Solin, *Nanoscale Adv*, 2023, **5**, 820-829.
9. T. Y. Liu, Y. R. Zheng, C. X. Hao, W. Q. Hong, F. X. Wang, H. Jang, Y. J. Hu and C. Z. Li, *Applied Materials Today*, 2023, **32**, 101801.
10. B. K. Tian, X. F. Jiang, W. C. Chu, C. X. Zheng, W. L. Guo and Z. H. Zhang, *Int J Smart Nano Mat.*, 2023, **14**, 230-242.
